# The impact of various calcium sources offered to Arabic hens during their early-laying stage on calcium consumption and egg production

**DOI:** 10.5455/javar.2023.j649

**Published:** 2023-03-31

**Authors:** Syafwan Syafwan, Agus Budiansyah, Ucop Haroen, Kristoper Simanungkalit, Lusia Agustina Br. Sembiring, Intan Lestari Aritonang

**Affiliations:** Animal Science Faculty, University of Jambi, Jambi, Indonesia

**Keywords:** Calcium, Arabic hens, choice feeding, egg, performance

## Abstract

**Objective:**

The research was conducted to calculate feed, calcium (Ca) intake, Ca requirements, and egg production for Arabic hens during the early egg-laying period.

**Materials and Methods:**

A total of 135 30-week-old Silver female Arabic pullets were randomly allocated to one of three treatments with five replicate cages with a semi-scavenging system of nine pullets per cage in a completely randomized design and allowed to choose Ca from limestone and oyster shells. As a control (T1), pullets were only given a complete feed with Ca and phosphorus percentages according to Hy-line International in 2018. Other treatment feeds were control feed without limestone fed with a combination of limestone (T2) or oyster shell (T3) separately.

**Results:**

The treatments had no effect (*p *> 0.05) on feed, grit and Ca intake (gm/bird/week), egg weight (gm), egg mass, egg production (%), and feed efficiency, but had an effect (*p < *0.05) on Ca concentration (%). Ca concentration was the same at T1 and T3, and both were higher than at T2.

**Conclusion:**

The female Arabic chickens could fulfill Ca requirements by selecting from different sources of Ca. Limestone is better than the oyster shell as a source of Ca. The Ca requirement for Arabic hens in the early laying period based on the Ca concentration of the feed intake is sufficient at around 3.64% since producing the same number of egg production and heavier egg weight compared to a higher Ca level.

## Introduction

Calcium (Ca) is one of the key compounds in egg shells, in expansion to its role in reducing the impact of high temperatures and maintaining bone health [1]. Egg production, egg weight, feed consumption, bone density, and strength and shell quality decrease when chickens are Ca deficient [2]. Furthermore, Additionally, poor shell quality will result in economic losses for farmers and producers because the eggshell is easily broken [3], even with adequate dietary Ca [4], and this frequently happens in the late spawning period, possibly as a result of disturbances related to Ca homeostasis [5].

Egg weight, shell weight, and plasma Ca concentration were lower on hens exposed to a temperature of 30°C than on hens exposed to a temperature of 18°C. Furthermore, the retention of several minerals, including Ca is lower in chickens reared at high cyclical ambient temperatures (24°C–35°C) than at 24°C [6]. This situation illustrates the Ca requirement can be higher in chickens at high temperatures than in chickens at low temperatures.

Since the greatest feed intake (FI) occurred at 14–16 h after lights-on, just before lights-out, the hourly FI pattern in laying hens is more closely tied to the alteration of night and day rhythm and is controlled by the egg arranging cycle. This greatest FI is undoubtedly strengthened by the shell design. In this manner, the more noteworthy utilization of Ca in conjunction with nourishing earlier the scotophase contributes to the supply of Ca for eggshell arrangement amid the night [7]. The increased weight of eggs and improved eggshell quality given with 3.5% Ca compared to 2.5% Ca level in the diet supported this phenomenon [8]. Increases in Ca intake on the day of ovulation may occur before and during the early stages of eggshell formation. When complete feeds are utilized, the hens’ intake is primarily regulated by their energy needs and the feed provided, but because they are unable to match their nutrient intake to their needs, the birds ingest excessive amounts of Ca to make up for the Ca needed to form eggshells. [7]. Therefore, a complete feed may not be suitable to ensure the Ca intake to fulfill the Ca requirement of the hens.

Numerous elements, including chicken strain, phosphorus, feed energy, chicken age, and temperature, have an impact on Ca requirements [5]. Chickens raised in seasonal settings and commercial chicken strains are typically addressed in the NRC 1994 literature or the Hy-Line Brown management guidelines [9] with regard to Ca requirements. The Ca requirements for layer chickens are negatively correlated with the FI and increase with the age of layer chickens due to the limited ability of animals to store Ca for egg formation and a slight increase in the amount of Ca deposited in the eggshell. Hy-line Brown-egg layers Ca requirement is 4.40% from 38 to 48 weeks of age [9] and Hy-line W36 varies from 3.61% to 4.37% for 95–115 gm FI from 33 to 55 weeks of age [10]. Commercial chickens’ Ca standard in seasonal areas may not be appropriate for Ca requirements of Indonesian chickens, particularly Arabic chickens raised in tropical climates. Chickens cannot maintain optimal shell quality even for one day without dietary Ca, due to the limited ability of chickens to store Ca for future shell formation [2].

Research on female Arabic chickens has recently been carried out to determine protein and energy requirements during the early egg production [11] and late egg production [12]. There has not been informed on Ca requirements for the Arabic chickens during the laying period. When the diet is in excess of Ca, Ca is excreted as Ca phosphate, leading to a phosphorous deficiency. A shortage of Ca in a layer hen’s diet can cause osteoporosis and weak leg bone and produces poorly-shelled eggs. In addition to affecting the availability of minerals including P, Mg, Mn, and Zn, high dietary Ca concentrations have also been linked to decreased phytase activity due to the creation of Caphytate [13,14]. Although it has been demonstrated that reducing dietary Ca levels decreases skeletal integrity, it has been reported that doing so increases the availability of phytate P [14]. In this way, decreasing dietary Ca concentrations may result in improved P digestibility and development execution, but may also harm the health and welfare of chickens through aggravated leg health concerns and reduced skeletal integrity [15]. William et al. [2] reported that with an average ambient temperature of 21.65°C, white-laying hens from 46 to 62 weeks of age required 3.56% of the diet or 4.0 gm of Ca per day. An et al. [5] indicated that aged brown layers require a substantially greater level of Ca (4.7%) to minimize cracked eggs and maximize eggshell quality compared to the amounts required by the current Korean feeding regulations for poultry (4.1%). In their study using H&N Brown Nick layers (60–72 weeks of age), Attia et al. [16] found that increasing the dietary Ca levels of laying hens by up to 4% during the late production period may be a useful strategy to improve eggshell quality, Haugh unit, laying performance, and physiological and immunological state. It is evident that the Ca need varies depending on breed, age, and temperature. As a result, it is difficult to define how much Ca Arabic laying chickens need in their diets.

A strategy to determine the nutrient requirements could be done by choice feeding because poultry appeared to have particular desires for supplements and can choose a count of calories from an assortment of sources to meet their wholesome prerequisites [11,12,15,17,18]. Choice feeding provides an opportunity for chickens to select different foods quickly to meet the nutritional needs of individuals during daily fluctuation caused by the temporal sequence of the egg development [7]. Because Arabic hens can consume Ca supplies when necessary, providing them in a separate feeder would be an appropriate technique for evaluating their needs during the egg-laying phase.

## Materials and Methods

### Ethical statement

All experiments received permission from the Animal Science Faculty’s Ethical Clearance Committee, reference number 003/UN21.7/ECC/2021. The house of chicken was half an open-sided house and the yard was available with free access at any time. There was no slaughter and blood withdrawal during the experiment.

### Animal care, birds, and housing

A total of 135 30-week-old Silver female Arabic pullets produced from Superior Livestock Breeding Center and Forage, Sembawa, South Sumatera were weighed and randomly housed in 15 pens with 9 pullets each. This chicken is a particular variety of native chicken raised for layers [19]. The pen was the same in width (1.14 m) and height (2 m) inside and outside the home, measuring 2 and 3 m, respectively. Each pen, which was divided with netting nylon, served as an experimental duplicate. The sand was spread across the pen floor within the house, and the ground was spread across the pen floor outside the house. The pullets had unrestricted access to the yard and the house had half open-sided.

In each pen, a different feeder delivered each diet. Every day, at random, the feeder locations in each pen were switched out to prevent the hens from becoming accustomed to them. When necessary, one bell-shaped drinker was filled in. The light was scheduled for 16:8 h light and dark.

### Experimental design and treatments

Three treatments and five replicates were used in the completely randomized design (CRD) of the study. The treatments applied were T1) control diet Crude Protein [CP = 18.9%, Metabolizable Energy (ME) = 30.43 kcal/kg, Ca = 4.2% and available phosphorus = 0.46%] (Table 1); T2), the control diet without limestone was fed with a combination of limestone separately and T3) the control diet without limestone was fed with a combination of oyster shell separately. All the diets were offered in mash form and ad libitum.

**Table 1.** The ingredients composition (%) and calculated nutrient content of dietary treatments.

Ingredients	Control
Rice bran	3.08
Maize	42.31
SBM (CP: 43.3%)	29.18
Fish meal (CP: 43.5%)	6.11
NaCl	0.21
Mineral feed suplement^a^	0.23
Dicalcium phosphate	0.75
Limestone	7.57
DL-Methionine	0.15
Palm oil	10.41
Total	100.00
Nutrient composition (Calculate)	
Dry matter (%)	78.76
CP (%)^b^	18.91
ME (Kkal/kg)^b,c^	3,049.01
Extract ether (%)	13.32
Crude fiber (%)	2.72
Lycine (%)	1.22
Methionine (%)	0.53
Met + Cys (%)	0.85
Ca (%)^d^	4.21
Total *P* (%)	0.83
Available phosphorous (%)	0.49
Na (%)^d^	0.19

a = Composition of 1 kg mineral feed supplement: calcium (Ca), 32.5%; phosphorus (P), 1.0%; iron (Fe), 6 gm; manganese (Mn), 4 gm; iodine (I), 0.075 gm, copper (Cu), 0.3 gm; zinc (Zn), 3.75 gm, vitamin B12 (cyanocobalamin), 0.5 mg and vitamin D3 (cholecalciferol), 50.000 IU.

b = Syafwan and Noferdiman [11].

c = Metabolizable energy was calculated by determining (combustion) gross energy of the entire diet multiplied with a ME to GE-conversion factor (0.725).

d = Hy-line Brown Commercial Management Guide (2018).

The percentage content of Ca and phosphorus (in the form of non-phytate phosphorus) in the control feed was compiled based on the recommendations of the Hy-line management guidelines [9]. The particle size distribution of the Ca source is 40% with a size of 0–2 mm and 60% with a size of 2–4 mm [9].

### Traits measured

Weighing the feed, grit, and residues each week (g/bird/week) allowed us to track the amount of feed (FI) and grit consumed each pen. The percentage of limestone in the diet was subtracted from the FI to determine the amount of mash consumed per pen in the control diet. The amount of Ca consumed was determined by multiplying it by the amount of Ca present in the feed and other sources of Ca. The amounts of Ca in the diet were computed by dividing the Ca intake by FI multiplied by 100 (%).

Daily records of egg weight and production were kept. The total number of eggs deposited each day divided by the total number of live hens was used to compute the percentage of hen day egg (HDP) production (% HDP) [11,20]. Egg mass (EM) was estimated by multiplying the average egg weight by the egg production percentage, and the feed conversion ratio (FCR) was calculated from FI by EM [21].

### Statistical analysis

Using SAS’ PROC MIXED, data were analyzed in accordance with the procedure outlined by Syafwan et al. [17]. Data for a mixed model with dietary treatment as the main effect and week as a repeated measurement were analyzed using the CRD. Since the data were collected weekly on the same animals, a mixed model utilizing SAS’s Mixed Procedure was utilized to ascertain the covariance structure among repeated observations [22,23]. The pen was taken into account as an additional random effect in the study, and the week was employed as the time component.

It was decided that a probability level of 5% qualified as statistically significant. When the main effects or their interactions were significant, means were compared using a pairwise comparison utilizing the Least Significant Difference. At the *p *< 0.05 level, the PDIFF option with the PDMIX800 SAS macro was used to separate the means of significant effects. [11,17,24]. The denominator df for the testing of main effects was calculated using the Kenward-Roger technique. The corrected Akaike Information Criteria served as the foundation for the best covariance structure. The best fit for FI was the unstructured covariance structure. For mash intake, grit intake, Ca intake, Ca concentration, and egg production, the simple covariance structure was the best match. The average egg weight fit well with the heterogeneous autoregressive covariance structure (1). For EM and FCR, the Autoregressive covariance structure (1) provided the best match.

## Results

Table 2 presents the probability values for all variables and Tables 3 and 4 show the performance of the pullets under different feeding methods. Feeding methods had no effect on FI and mass intake of the pullets (Table 2), although FI and mash intake fluctuation occurred for some weeks. During the first 2 weeks, FI and mash intake of control-fed pullets were below but above the two counterparts during 33–41 weeks, respectively (Table 3). FI and mash intake of limestone-fed pullet below and above the oyster-fed pullet during 32–35 weeks and 36–41 weeks, respectively. The control pullets and the self-selection pullets consumed almost the same amount of feed on average (*p *> 0.05). A self-selection limestone and the self-selection oyster pullets consumed numerically less mash than their control counterparts (*p *> 0.05; Table 2).

Feeding methods affected grit intake, Ca intake, and Ca concentration (Table 2). Grit intake (*p *< 0.001), Ca intake (*p *< 0.001), and Ca concentration (*p *< 0.001) were lower in the choice-fed pullets (Table 2). The grit intake of the control-fed pullets was significantly higher than its two counterparts for most of the week (Table 3). Those two self-selection-fed pullets, started to increase the grit intake from 32 to 38 weeks of age and slightly decrease from 39 weeks onward, but still above the grit intake at 31 weeks of age. Therefore, the inclusion of grit intake did not bring a significant effect on total FI on self-selection-fed hens. Lower grit intake despite equal total FIs indicates that mash consumption was high for self-selection-fed hens.

The control-fed pullets always increased in Ca intake from 33 weeks of age onward and limestone self-selection-fed hens were always low in Ca intake every week. However, the Ca intake of oyster shell self-selection pullets increased from 31 to 34 weeks of age and decreased from 35 to 41 weeks of age. The significantly lower Ca intake of limestone self-selection-fed hens than two counterparts started from 37 to 41 weeks of age (Table 3). On average, the Ca intake for control-fed hens (23.96 gm) was similar to oyster shell self-selection-fed hens (23.26 gm) and limestone self-selection-fed hens (19.95 gm) was lower than the two counterparts (*p *< 0.05; Table 2).

The Ca concentration in the diet consumed for limestone self-selection-fed hens was always below control-fed and oyster shell self-selection pullets every week. From 31 to 38 weeks of age, oyster shell self-selection pullets’ Ca content was higher than that of control-fed pullets, and afterwards it was lower. However, the Ca concentration in the diet consumed for limestone self-selection-fed hens was significantly lower than control-fed pullets and oyster self-selection-fed hens most of the week (Table 3). Overall, the Ca concentration for oyster shell self-selection pullets (4.27%) and control pullets (4.20%) were similar but they were significantly higher than limestone self-selection-fed hens (3.64%; *p *< 0.05; Tables 2 and 3).

**Figure 1.**
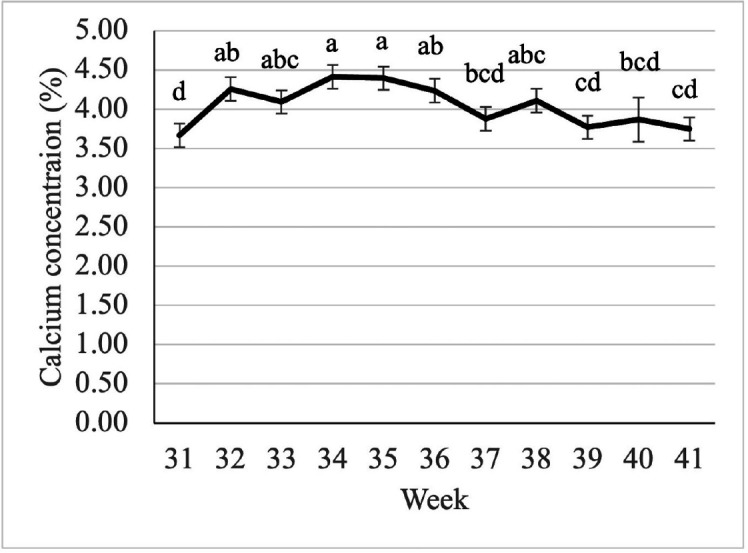
Least square means for calcium concentration shows a significant week effect.Means without a common letter (a–d) differ significantly (*p* < 0.05).

Week had an impact on Ca levels (*p *< 0.01), but there was no interaction between week and feeding method (*p* > 0.05). From the 32nd to the 36th week of age, the Ca concentration greatly rose, and from the 39th to the 41st week of age, the Ca concentration significantly decreased (Fig. 1).

The pullets’ egg production was unaffected by the feeding technique (*p *> 0.05; Table 2). The week had no effect on egg production, and there was no interaction between week and feeding technique (Table 2). During the first 3 weeks of the study, egg production of control-fed pullets was low and turned above the other counterparts from the 35th to 41st week of age. The egg production of limestone self-selection-fed hens was always above the egg production of oyster self-selection-fed hens from the 36th to 41st week of age. Therefore, the egg production of oyster and limestone self-selection-fed hens was numerically low and high, respectively (Table 4).

**Table 2.** Probability values^a^ of main effects and interaction between feeding method (*F*)^b^ and week for different variables.

Main effect	T1	T2	T3	*F*	Week	F*Week
FI (gm/hen/week)	570.56	552.61	543.95	0.600	0.056	0.130
Grit intake (gm/hen/week)	43.19	34.84	30.48	**<0.001**	0.121	0.342
Mash intake (gm/hen/week)	527.37	517.77	513.48	0.798	0.058	0.830
Calcium intake (gm/hen/week)	23.96	19.95	23.26	**<0.001**	0.192	0.405
Calcium concentration (%/hen/week)	4.20	3.64	4.27	**<0.001**	**0.001**	0.318
Egg production (%)	62.70	63.73	60.67	0.326	0.914	0.090
Egg weight (kg/hen/week)	1.66	1.62	1.54	**0.048**	0.323	**0.009**
Average egg weight (gm)	43.29	43.68	43.02	0.491	**0.001**	0.995
EM	27.20	27.86	26.01	0.612	0.064	0.372
FCR	3.06	2.91	3.07	0.591	**0.021**	0.388

Note: ^a^Probability values with boldface differ significantly (*p *≤ 0.05).

^b^T1) control diet (CP = 18.9%, ME = 30.43 kcal/kg, calcium = 4.2% and available phosphorus = 0.46%) (Table 1); T2), the control diet without limestone and limestone separately and T3) the control diet without limestone and oyster shell separately.

The bold value is to emphasize significant differences (*p* < 0.5).

**Table 3.** Least square means of intake performance variables in Arabic pullets from 31 to 41 weeks of age as affected by feeding method^ a^

Variables	Treatments	Week					
		31	32	33	34	35	36
FI (gm/hen/week)	T1 (Control)	543.31 ± 32.25	491.24 ± 18.79	565.47 ± 14.57	555.96 ± 17.81	557.44 ± 19.93	561.06 ± 20.49
	T2 (Limestone)	553.97 ± 36.05	500.64 ± 21.01	527.28 ± 16.29	542.97 ± 19.91	517.56 ± 22.29	535.23 ± 22.91
	T3 (Oyster shell)	538.58 ± 32.25	525.48 ± 18.79	539.49 ± 14.57	555.99 ± 17.81	540.91 ± 19.93	522.76 ± 20.49
	Probability	*p* > 0.05	*p* > 0.05	*p* > 0.05	*p* > 0.05	*p* > 0.05	*p* > 0.05
Mash intake (gm/hen/week)	T1 (Control)	502.18 ± 29.51	454.06 ± 17.10	522.66 ± 14.20	513.87 ± 16.19	515.25 ± 20.07	518.59 ± 19.53
	T2 (Limestone)	524.22 ± 32.99	465.14 ± 19.11	491.92 ± 15.87	501.56 ± 18.11	479.33 ± 22.44	494.11 ± 21.84
	T3 (Oyster shell)	515.18 ± 29.51	492.37 ± 17.10	509.11 ± 14.20	518.34 ± 16.19	504.87 ± 20.07	492.78 ± 19.53
	Probability	*p* > 0.05	*p* > 0.05	*p* > 0.05	*p* > 0.05	*p* > 0.05	*p* > 0.05
Grit intake (gm/hen/week)	T1 (Control)	41.13 ± 3.43	37.19 ± 3.43	42.81 ± 3.43	42.09 ± 3.43	42.20 ± 3.43	42.47 ± 3.43
	T2 (Limestone)	29.75 ± 3.83	35.50 ± 3.83	35.36 ± 3.83	41.42 ± 3.83	38.22 ± 3.83	41.12 ± 3.83
	T3 (Oyster shell)	23.40 ± 3.43	33.12 ± 3.43	30.38 ± 3.43	37.66 ± 3.43	36.03 ± 3.43	29.98 ± 3.43
	Probability	***p* < 0.05**	*p* > 0.05	***p* < 0.05**	*p* > 0.05	*p* > 0.05	***p* < 0.05**
Calcium intake (gm/hen/week)	T1 (Control)	22.82 ± 1.80	20.63 ± 1.80	23.75 ± 1.80	23.35 ± 1.80	23.41 ± 1.80	23.56 ± 1.80
	T2 (Limestone)	17.98 ± 2.02	19.60 ± 2.02	19.85 ± 2.02	22.38 ± 2.02	20.97 ± 2.02	22.18 ± 2.02
	T3 (Oyster shell)	19.26 ± 1.80	24.51 ± 1.80	23.15 ± 1.80	27.39 ± 1.80	26.31 ± 1.80	22.74 ± 1.80
	Probability	*p* > 0.05	*p* > 0.05	*p* > 0.05	*p *> 0.05	*p* > 0.05	*p* > 0.05
Calcium concentration (%)	T1 (Control)	4.20 ± 0.25	4.20 ± 0.25	4.0 ± 0.25	4.20 ± 0.25	4.20 ± 0.25	4.20 ± 0.25
	T2 (Limestone)	3.23 ± 0.28	3.92 ± 0.28	3.78 ± 0.28	4.12 ± 0.28	4.11 ± 0.28	4.13 ± 0.28
	T3 (Oyster shell)	3.56 ± 0.25	4.64 ± 0.25	4.29 ± 0.25	4.92 ± 0.25	4.86 ± 0.25	4.37 ± 0.25
	Probability	***p* < 0.05**	*p* > 0.05	*p* > 0.05	***p* < 0.05**	***p* < 0.05**	*p* > 0.05
FI (gm/hen/week)	T1 (Control)	600.87 ± 41.85	590.26 ± 27.85	635.09 ± 43.71	562.20 ± 49.37	613.29 ± 38.92	570.56 ± 18.48
	T2 (Limestone)	572.46 ± 46.79	593.07 ± 31.14	590.77 ± 48.87	588.37 ± 55.19	556.38 ± 43.51	552.61 ± 20.67
	T3 (Oyster shell)	567.64 ± 41.85	566.57 ± 27.85	569.46 ± 43.71	530.17 ± 49.37	526.45 ± 38.92	543.95 ± 18.48
	Probability	*p *> 0.05	*p *> 0.05	*p *> 0.05	*p *> 0.05	*p *> 0.05	*p *> 0.05
Mash intake (gm/hen/week)	T1 (Control)	555.38 ± 40.40	545.58 ± 26.19	587.02 ± 40.30	519.64 ± 48.43	566.87 ± 36.20	527.37 ± 14.82
	T2 (Limestone)	541.48 ± 45.16	557.74 ± 29.28	558.23 ± 45.06	554.79 ± 54.14	526.94 ± 40.47	517.77 ± 16.57
	T3 (Oyster shell)	537.46 ± 40.40	531.12 ± 26.19	541.97 ± 40.30	504.21 ± 48.43	500.86 ± 36.20	513.48 ± 14.82
	Probability	*p *> 0.05	*p *> 0.05	*p *> 0.05	*p *> 0.05	*p *> 0.05	*p *> 0.05
Grit intake (gm/hen/week)	T1 (Control)	45.49 ± 3.43	44.68 ± 3.43	48.08 ± 3.43	42.56 ± 3.43	46.43 ± 3.43	43.19 ± 1.03
	T2 (Limestone)	30.98 ± 3.83	35.33 ± 3.83	32.54 ± 3.83	33.57 ± 3.83	29.44 ± 3.83	34.84 ± 1.15
	T3 (Oyster shell)	30.18 ± 3.43	35.46 ± 3.43	27.49 ± 3.43	25.96 ± 3.43	25.60 ± 3.43	30.48 ± 1.03
	Probability	***p* < 0.05**	*p *> 0.05	***p* < 0.05**	***p* < 0.05**	***p* < 0.05**	***p* < 0.05**
Calcium intake (gm/hen/week)	T1 (Control)	25.24 ± 1.80	24.79 ± 1.80	26.67 ± 1.80	23.61 ± 1.80	25.76 ± 1.80	23.96 ± 0.54
	T2 (Limestone)	18.67 ± 2.02	20.60 ± 2.02	19.49 ± 2.02	19.86 ± 2.02	17.89 ± 2.02	19.95 ± 0.61
	T3 (Oyster shell)	23.37 ± 1.80	26.29 ± 1.80	21.89 ± 1.80	20.59 ± 1.80	20.34 ± 1.80	23.26 ± 0.54
	Probability	***p* < 0.05**	***p* < **0.05	***p* < 0.05**	*p *> 0.05	***p* < 0.05**	***p* < 0.05**
Calcium concentration (%)	T1 (Control)	4.20 ± 0.25	4.20 ± 0.25	4.20 ± 0.25	4.20 ± 0.25	4.20 ± 0.25	4.20 ± 0.08
	T2 (Limestone)	3.24 ± 0.28	3.45 ± 0.28	3.31 ± 0.28	3.47 ± 0.28	3.27 ± 0.28	3.64 ± 0.08
	T3 (Oyster shell)	4.18 ± 0.25	4.67 ± 0.25	3.79 ± 0.25	3.92 ± 0.25	3.77 ± 0.25	4.27 ± 0.08
	Probability	***p* < 0.05**	***p* < 0.05**	***p* < 0.05**	*p *> 0.05	***p* < 0.05**	***p* < 0.05**

^a^T1) control diet (CP = 18.9%, ME = 30.43 kcal/kg, calcium = 4.2% and available phosphorus = 0.46%) (Table 1); T2), the control diet without limestone and limestone separately and T3) the control diet without limestone and oyster shell separately.

The bold value is to emphasize significant differences (*p* < 0.5).

The egg weight was impacted by the feeding technique (*p *< 0.05; Table 2). From the 35th to 41st week of age, oyster self-selection-fed hens’ egg weight was significantly lower than that of control-fed pullets, which was low from the 31st to 34th week of age (Table 4). From the 31st to the 41st week of age, the egg weight of the limestone self-selection-fed hens was comparable to that of the control and oyster self-selection-fed hens. However, the egg weight of oyster self-selection-fed hens was significantly lower than control-fed pullets from the 38th to 41st week of age. Overall, the egg weight of control-fed pullets was higher than oyster self-selection-fed hens but similar to limestone self-selection-fed hens, and the egg weight of the limestone self-selection-fed hens was not significantly higher than oyster self-selection-fed hens (Table 4).

**Figure 2.**
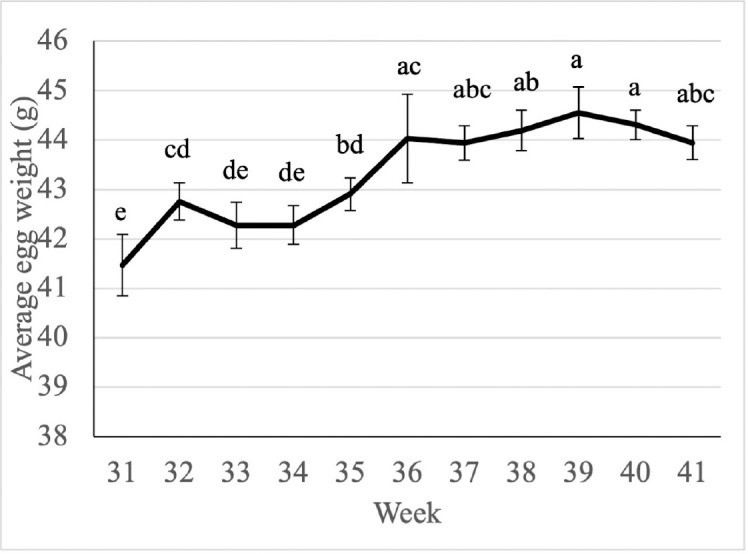
Least square means for average egg weight shows a significant week effect. Means without a common letter (a–d) differ significantly (*p* < 0.05).

The average egg weight was unaffected by the feeding method, and there was no interaction between it and the week (*p* > 0.05; Table 2). With the exception of the 34th, 35th, and 39th weeks, control-fed pullets’ average egg weight was consistently in the middle, and limestone self-selection-fed birds’ average egg weight was consistently above average. For the most of the week, oyster self-selection-fed hens’ average egg weight was consistently low (Table 4). The average egg weight varied by the week (*p* = 0.001). From the 36th week of age until the completion of the trial, the average egg weight increased dramatically every week and was higher than before (Fig. 2). This means that the weight of the egg was heavy when the pullets become older.

**Figure 3.**
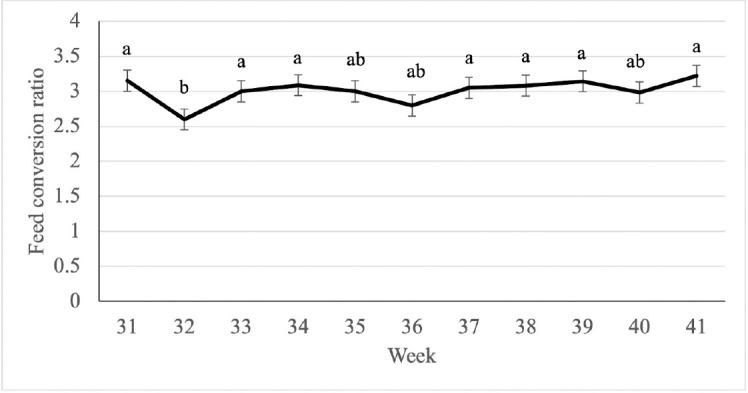
Least square means for the FCR shows a significant week effect. Means without a common letter (a–b) differ significantly (*p* < 0.05).

The feeding method, week, or interaction had no effect on EM (*p* > 0.05; Table 2). The week had an impact on the FCR but not the feeding strategy (*p* = 0.023; Table 2). FCR dramatically increased on the 33rd week of age after having significantly fallen significantly on the 32nd week of age, and remained steady for the remainder of the research (Fig. 3). The feeding strategy and the week on FCR did not interact (*p* > 0.05; Table 2).

## Discussion

The FI was similar between feeding treatments and perhaps it was due to the energy and protein content of each feed in each treatment being the same and the appetite of the feed is similar, the only difference being the Ca content of each treatment. The energy content of the feed was 3049.01 kcal ME/kg and protein was 18.91%. If the energy and protein contents in the feed are sufficient and balanced, it will have the same effect on the consumption of the feed. Therefore, the FI is adjusted to ensure a sufficient energy demand in the long term for growth and egg production. In response to a continually changing external environment, adaptive adjustments in FI, digestion, absorption, and metabolism work together to maintain whole-body energy balance and BW [25].

The consumption of Ca on limestone self-selection-fed birds was lower (*p *< 0.001) than that of control-fed birds and oyster shell self-selection-fed birds. This shows that the chicken is trying to meet its needs by adjusting the Ca that is suitable for them. According to Syafwan et al. [18], the provision of selective feeding provides the opportunity for birds to choose the type of feed they prefer, especially regarding the fulfillment of nutrients based on their physiological needs.

Ca concentration in limestone self-selection-fed birds was significantly lower than in control and oyster shell self-selection-fed birds. The lower Ca concentration in limestone self-selection-fed birds was due to a significantly lower Ca intake and a numerically lower percentage of Ca in the limestone than in oyster shells. These results may suggest that the concentration of Ca supplied in brown-layer chicken diets (4.2% in the control diet) is higher than that of the Ca requirement for Arabic hens. However, when the oyster shell was a source of Ca, the Ca concentration in the feed consumed was as much as the Ca concentration in the control diet. The laying hens in our study have a long period to learn and to make the appropriate choices from feeding regimens and are more adaptable. It seems that there was a different appetite between limestone and oyster shells. Since the birds needed visual or gustatory cues to distinguish between the diets, it is possible that the specific hunger for Ca is driven by a diet-related characteristic rather than by Ca itself [26]. William et al. [2] reported that with an average ambient temperature of 21.65°C, white-laying hens from 46 to 62 weeks of age required 3.56% of the diet or 4.0 gm of Ca per day. An et al. [5] reported that aged brown layers need a significantly higher Ca level (4.7%) than what is currently permitted by Korean feeding standards for chicken(4.1%) in order to reduce cracked eggs and maximize eggshell quality. Attia et al. [16] found that increasing the dietary Ca levels of laying hens by up to 4% during the late production period (60–72 weeks of age) may be a useful strategy to improve eggshell quality, Haugh unit, laying performance, and physiological and immunological state in H&N Brown Nick layers. In addition, hens’ performance was improved in the late stages of production when 3.5% Ca diets were supplemented with 1,000 IU of vitamin D3 or 4,000 IU/kg of diet vitamin D3, proving that vitamin D3’s effects are reliant on dietary Ca concentrations. According to Lichovníková and Zeman [27], laying hens’ housing system determines the Ca requirement. The eggshell strength of the hens housed in the floor system was both the weakest and much lower than that of the cage systems. The hens utilized Ca more effectively in the cage systems compared to the floor system, resulting in higher-quality eggshells. The hens in the unenriched cage had the highest Ca requirements due to the system’s maximum eggshell output and quality. However, the tibia had the weakest strength at that location. The housing system should be considered when determining how much Ca is needed in laying hens’ feed. In spite of the fact that although both the hens in the floor system and those in enhanced cages consumed Ca, the floor system’s eggshells had less of it. According to Kismiati et al. [28], the Ca sources had an impact on how well laying hens produced eggs. When used as a Ca source for feed, eggshell waste performed better than limestone or a combination of limestone and eggshell waste. As a result, a variety of variables, including Ca sources, hen ages, temperatures, the quality of the eggshell, and housing system, affect the Ca requirement.

**Table 4.** Least square means of egg performance variables in Arabic pullets from 31 to 41 weeks of age as affected by feeding method^a^

Variables	Treatments	Week					
		31	32	33	34	35	36
Egg production (%)	T1 (Control)	55.87 ± 4.66	60.32 ± 4.66	55.24 ± 4.66	59.68 ± 4.66	65.51 ± 4.66	63.27 ± 4.66
	T2 (Limestone)	66.67 ± 5.21	70.63 ± 5.21	65.87 ± 5.21	57.54 ± 5.21	54.06 ± 5.21	65.00 ± 5.21
	T3 (Oyster shell)	60.63 ± 4.66	70.71 ± 4.66	68.10 ± 4.66	67.26 ± 4.66	63.79 ± 4.66	61.31 ± 4.66
	Probability	*p *> 0.05	*p *> 0.05	*p *> 0.05	*p *> 0.05	*p *> 0.05	*p *> 0.05
Egg weight (kg/hen/week)	T1 (Control)	1.45 ± 0.12	1.62 ± 0.12	1.47 ± 0.12	1.57 ± 0.12	1.73 ± 0.12	1.67 ± 0.12
	T2 (Limestone)	1.76 ± 0.13	1.94 ± 0.13	1.76 ± 0.13	1.55 ± 0.13	1.45 ± 0.13	1.55 ± 0.13
	T3 (Oyster shell)	1.57 ± 0.12	1.87 ± 0.12	1.77 ± 0.12	1.75 ± 0.12	1.68 ± 0.12	1.55 ± 0.12
	Probability	*p *> 0.05	*p *> 0.05	*p *> 0.05	*p *> 0.05	*p *> 0.05	*p *> 0.05
Average egg weight (gm/egg)	T1 (Control)	41.27 ± 1.04	42.41 ± 0.63	42.16 ± 0.78	41.70 ± 0.65	42.15 ± 0.55	43.96 ± 1.49
	T2 (Limestone)	41.94 ± 1.16	43.65 ± 0.70	42.55 ± 0.87	42.82 ± 0.73	43.39 ± 0.61	43.66 ± 1.67
	T3 (Oyster shell)	41.18 ± 1.04	42.20 ± 0.63	42.11 ± 0.78	42.31 ± 0.65	43.19 ± 0.55	44.46 ± 1.49
	Probability	*p *> 0.05	*p *> 0.05	*p *> 0.05	*p *> 0.05	*p *> 0.05	*p *> 0.05
EM	T1 (Control)	23.05 ± 2.10	25.64 ± 2.10	23.29 ± 2.10	24.88 ± 2.10	27.60 ± 2.10	27.74 ± 2.10
	T2 (Limestone)	27.87 ± 2.35	30.78 ± 2.35	27.97 ± 2.35	24.68 ± 2.35	23.46 ± 2.35	28.50 ± 2.35
	T3 (Oyster shell)	24.87 ± 2.10	29.83 ± 2.10	28.65 ± 2.10	28.44 ± 2.10	27.53 ± 2.10	27.00 ± 2.10
	Probability	*p *> 0.05	*p *> 0.05	*p *> 0.05	*p *> 0.05	*p *> 0.05	*p *> 0.05
FCR	T1 (Control)	3.40 ± 0.25	2.84 ± 0.25	3.59 ± 0.25	3.24 ± 0.25	2.94 ± 0.25	2.86 ± 0.25
	T2 (Limestone)	2.89 ± 0.28	2.32 ± 0.28	2.71 ± 0.28	3.23 ± 0.28	3.21 ± 0.28	2.96 ± 0.28
	T3 (Oyster shell)	3.17 ± 0.25	2.63 ± 0.25	2.71 ± 0.25	2.79 ± 0.25	2.85 ± 0.25	2.58 ± 0.25
	Probability	*p *> 0.05	*p *> 0.05	***p* < 0.05**	*p *> 0.05	*p *> 0.05	*p *> 0.05
Egg production (%)	T1 (Control)	64.04 ± 4.66	66.30 ± 4.66	66.98 ± 4.66	66.49 ± 4.66	65.99 ± 4.66	62.70 ± 1.41
	T2 (Limestone)	64.84 ± 5.21	63.89 ± 5.21	65.24 ± 5.21	65.16 ± 5.21	62.14 ± 5.21	63.73 ± 1.57
	T3 (Oyster shell)	59.72 ± 4.66	56.90 ± 4.66	53.77 ± 4.66	53.73 ± 4.66	51.39 ± 4.66	60.67 ± 1.41
	Probability	*p *> 0.05	*p *> 0.05	***p* < 0.05**	*p *> 0.05	***p* < 0.05**	*p *> 0.05
Egg weight (kg/hen/week)	T1 (Control)	1.69 ± 0.12	1.76 ± 0.12	1.83 ± 0.12	1.79 ± 0.12	1.75 ± 0.12	1.66 ± 0.04
	T2 (Limestone)	1.55 ± 0.13	1.53 ± 0.13	1.58 ± 0.13	1.61 ± 0.13	1.53 ± 0.13	1.62 ± 0.04
	T3 (Oyster shell)	1.45 ± 0.12	1.40 ± 0.12	1.31 ± 0.12	1.33 ± 0.12	1.24 ± 0.12	1.54 ± 0.04
	Probability	*p *> 0.05	***p* < 0.05**	***p* < 0.05**	***p* < 0.05**	***p* < 0.05**	***p* < 0.05**
Average egg weight (gm/egg)	T1 (Control)	44.12 ± 0.58	44.25 ± 0.68	45.41 ± 0.86	44.60 ± 0.50	44.26 ± 0.56	43.30 ± 0.39
	T2 (Limestone)	44.08 ± 0.65	44.60 ± 0.76	44.74 ± 0.96	44.69 ± 0.55	44.34 ± 0.63	43.68 ± 0.44
	T3 (Oyster shell)	43.62 ± 0.58	43.72 ± 0.68	43.51 ± 0.86	43.64 ± 0.50	43.24 ± 0.56	43.02 ± 0.39
	Probability	*p *> 0.05	*p *> 0.05	*p *> 0.05	*p *> 0.05	*p *> 0.05	*p *> 0.05
EM	T1 (Control)	28.22 ± 2.10	29.39 ± 2.10	30.43 ± 2.10	29.73 ± 2.10	29.28 ± 2.10	27.20 ± 1.26
	T2 (Limestone)	28.66 ± 2.35	28.68 ± 2.35	29.28 ± 2.35	29.07 ± 2.35	27.47 ± 2.35	27.86 ± 1.41
	T3 (Oyster shell)	26.02 ± 2.10	24.81 ± 2.10	23.33 ± 2.10	23.48 ± 2.10	22.13 ± 2.10	26.01 ± 1.26
	Probability	*p *> 0.05	*p *> 0.05	*p *> 0.05	*p *> 0.05	*p *> 0.05	*p *> 0.05
FCR	T1 (Control)	3.12 ± 0.25	2.90 ± 0.25	2.98 ± 0.25	2.74 ± 0.25	3.04 ± 0.25	3.06 ± 0.12
	T2 (Limestone)	2.85 ± 0.28	3.03 ± 0.28	2.90 ± 0.28	2.92 ± 0.28	2.92 ± 0.28	2.91 ± 0.13
	T3 (Oyster shell)	3.18 ± 0.25	3.31 ± 0.25	3.54 ± 0.25	3.29 ± 0.25	3.70 ± 0.25	3.07 ± 0.12
	Probability	*p *> 0.05	*p *> 0.05	*p *> 0.05	*p *> 0.05	*p *> 0.05	*p *> 0.05

^a^T1) control diet (CP = 18.9%, ME = 30.43 kcal/kg, calcium = 4.2% and available phosphorus = 0.46%) (Table 1); T2), the control diet without limestone and limestone separately and T3) the control diet without limestone and oyster shell separately.

The bold value is to emphasize significant differences (*p* < 0.5).

The house system in our study was semi-scavenging and the hens had free access to the yard day and night. The advantages of a semi-scavenging system are the hens can spend their time outside the house to express their behavior when the temperature is suitable for them and could get sunlight. The skin of the hens may have begun to process the synthesis of vitamin D when they were exposed to sunshine. The production of vitamin D in the skin, which is affected by UV light intensity and changes with latitude and season, is the most significant source of vitamin D. DBP, or vitamin D binding protein, carries vitamin D from the bloodstream to the liver, where it links serum levels of vitamin D and its metabolites. When vitamin D is hydroxylated at C-25 in the liver, 25-hydroxyvitamin D3 (25(OH)D3) is produced. The main form of vitamin D that circulates, 25(OH)D3, is one of the most reliable indicators of vitamin D status. [29]. The Ca-binding protein, which is involved in the active transport of Ca across the intestinal wall, needs vitamin D3 to function [16]. The integration of the metabolism of Ca, phosphorus, and vitamin D3 is crucial for the development of eggshells and the wellbeing of bone in laying hens [30]. According to Rodriguez-Lecompte et al. [31], 25-(OH)D, the active form of vitamin D3, and vitamin D3 both show potent immunomodulatory activities with the support of T cells (Th2) over time. We assumed that the amount of Vitamin D in the mineral feed supplement used (Table 1) plus vitamin D3 produced by the synthesis from the skin on limestone-fed hens was sufficient to produce the egg although the Ca concentration was lower. This result was in line with the report of Attia et al. [16] that the supplementation of vitamin D3 at 1,000–1,200 IU/kg diet in the basal diet containing 3,000 IU/kg was more pronounced in a lower Ca diet (3.5% to 4.0%) than in a high Ca diet (4.5%) for the laying rate.

The significantly higher egg weight of the control-fed hen and limestone self-selection-fed hen than oyster self-selection-fed hen (Table 2) due to the lower egg weight of the oyster self-selection-fed hen during the last 4 weeks of the study. This phenomenon could be influenced by a large decrease in egg production (about 8.3%) of oyster self-selection-fed hens from the 37th to 41st week of age compared with a small decrease (about 2.7%) in egg production of limestone self-selection-fed hens while almost constant in egg production of control-fed hens.

Egg production, average egg weight, EM, and FCR were not significantly impacted by the feeding technique (*p* > 0.05). These results implied that the amount of Ca intake on limestone self-selection fed hen could optimize for Arabic hen resulting in the same number of egg production with a higher Ca intake as at control and oyster shell self-selection fed hen. Accordingly, the Ca content of about 3.64% was enough to meet the needs of Arabic chickens for Ca throughout the early stages of egg production because they produced the same percentage of egg production as a hen which consumes a higher Ca concentration (control and oyster shell self-selection fed birds). It has been demonstrated that adding a lot of Ca to poultry diets raises the pH of the bird digesta and gizzards [32]. Therefore, reducing dietary Ca lowers intestinal pH, reduces the formation of Ca phosphate precipitates, and increases pepsin availability, thus improving nutrient digestibility in poultry diets [33]. Our results were not completely different from Wilkinson et al. [26], who conducted a review, choice-fed hens had better average egg weight, shell thickness, and feed efficiency than hens on a traditional mash diet. Production of eggs did not differ, though. The results from this study showed that the requirement of Ca of about 3.64% was sufficient for Arabic hens during early egg production although it did not significantly lower the FCR. However, these values of dietary Ca requirements of Arabic hens are lower than the value recommended by Hy-Line of about 4.2% [9] for brown chicken, which would be of great significance for reducing the feed cost and Ca excretion.

In our study, Ca requirements were considered mostly for preventing Ca deficiency. In the future, A comprehensive approach should be used to determine the precise Ca requirement for Arabic hens. This approach takes into account not only health and metabolic endpoints but also the regulation of metabolic pathways by using noninvasive variables such as bone mineral content, bone mineral density, and eggshell Ca content.

## Conclusion

The female Arabic chickens could fulfill Ca requirements by selecting from different sources of Ca. Limestone is better than the oyster shell as a source of Ca. The Ca requirement for Arabic hens in the early laying period based on the Ca concentration of the FI is sufficient at around 3.64% since producing the same number of egg production and heavier egg weight compared to a higher Ca level.

## List of Abbreviations

BW, Body Weight; Ca, Calcium; CP, Crude protein; CRD, Completely randomized design; DBP, vitamin D Binding Protein; EM, Egg mass; FCR, Feed conversion ratio; FI, Feed intake; HDP, Hen day egg production; ME, Metabolizable energy; PDIFF, P- value for DIFFerences; SAS, Statistical Analysis System
